# Antimicrobial photodynamic therapy mediated by methylene blue and potassium iodide to treat urinary tract infection in a female rat model

**DOI:** 10.1038/s41598-018-25365-0

**Published:** 2018-05-08

**Authors:** Ying-Ying Huang, Anton Wintner, Patrick C. Seed, Timothy Brauns, Jeffrey A. Gelfand, Michael R. Hamblin

**Affiliations:** 1 Wellman Center for Photomedicine, Massachusetts General Hospital, Boston, MA 02114 USA; 2 Department of Dermatology, Harvard Medical School, Boston, MA 02115 USA; 3 Department of Urology, Massachusetts General Hospital, Boston, MA 02114 USA; 4 Department of Pediatrics, Duke University Medical Center, Durham, NC USA; 5 Department of Medicine, Massachusetts General Hospital, Boston, MA 02114 USA; 6 Harvard-MIT Division of Health Sciences and Technology, Cambridge, MA 02139 USA

## Abstract

Drug-resistant urinary tract infections (UTIs) are difficult and sometimes impossible to treat. Many UTIs are caused by uropathogenic *Escherichia coli* (UPEC). We developed an intact rat model of UTI, by catheterizing female rats and introducing a bioluminescent UPEC strain into the female rat bladder which lasted for up to six days. We recently showed that antimicrobial photodynamic inactivation (aPDI) of a bacterial infection mediated by the well-known phenothiazinium salt, methylene blue (MB) could be strongly potentiated by addition of the non-toxic salt potassium iodide (KI). In the intact rat model we introduced MB into the bladder by catheter, followed by KI solution and delivered intravesicular illumination with a diffusing fiber connected to a 1 W 660 nm laser. Bioluminescent imaging of the bacterial burden was carried out during the procedure and for 6 days afterwards. Light-dose dependent loss of bioluminescence was observed with the combination of MB followed by KI, but recurrence of infection was seen the next day in some cases. aPDT with MB + KI gave a significantly shorter duration of infection compared to untreated controls. aPDT with MB alone was the least effective. No signs of aPDT damage to the bladder lining were detected. This procedure to treat urinary tract infections without antibiotics by using already approved pharmaceutical substances (MB and KI) may have clinical applicability, either initially as a stand-alone therapy, or as an adjunct to antibiotic therapy by a rapid and substantial reduction of the bacterial burden.

## Introduction

Catheter-associated urinary tract infections (CAUTIs) represent a significant challenge for U.S. medicine, with ~ million CAUTIs /year in people who must self-catheterize, have long-term indwelling catheters, and those catheterized during acute medical care^1–3^. For millions with neurogenic bladder, for which repeated self-catheterization is needed, CAUTIs, generate hundreds of thousands of outpatient visits, ER visits or hospital admissions annually^1,4^. Within health care facilities (including long term care facilities where up to 10% of residents are chronically catheterized^5,6^), the costs of CAUTIs are hundreds of millions of dollars and >13,000 deaths^7,8^. Standard treatment for CAUTIs involves removal/change of catheter and antibiotics. For patients requiring repeated or chronic catheterization, the development of recurrent, symptomatic infections means repeated courses of antibiotic therapy, problematic due to the increasing prevalence of multidrug-resistant (MDR) organisms in UTIs and CAUTIs in particular. Extended-spectrum beta-lactamase (ESBL) producing *Escherichia coli* and *Klebsiellae* are emerging globally within healthcare facilities^9–11^ as well as in the community^12,13^, characterized by resistance to all penicillins, cephalosporins and aztreonam, often cross-resistant to trimethoprim/ sulfamethoxazole and quinolones^11^. Increased prevalence of carbapenem-resistant enterobacteria (CRE) has been reported in health care facilities in both the United States and abroad, the majority being Klebsiellae^14,15^. Both ESBL and CRE CAUTIs are linked to more complications, prolonged hospitalizations and significantly increased costs of care^9^. In fact, chronic CAUTIs are one of the leading reservoirs of MDR organisms in health-care institutions. Recurrent antibiotic therapy also exposes patients to the risk of *Clostridium difficile* associated diarrhea and antibiotic hypersensitivities, all attendant with additional morbidity and even mortality. As *E. coli* and *Klebsiella* are the most frequent cause of CAUTIs diagnosed in U.S. healthcare facilities^16^, these patterns of drug resistance are of great concern. Studies of the increased prevalence of ESBL-*E. coli* have identified a number of predisposing risk factors, but a key factor is the use of antibiotics^17–19^.

Antimicrobial photodynamic therapy (aPDT), a specific form of PDT in general, is the term used to describe the combination of non-toxic dyes called photosensitzers (PS) and light that in the presence of oxygen produces highly reactive oxygen species (ROS) such as singlet oxygen (^1^O_2_, Type II photochemical mechanism) and hydroxyl radicals (HO·, Type I photochemical mechanism)^20^. These ROS can damage biomolecules (proteins, lipids, nucleic acids) in a wide range of microorganisms regardless of structure or drug resistance and produce rapid killing of many logs of cells. If light is delivered soon after introduction of PS into infected tissue, significant selectivity for microbial cells over host cells is achieved^21^. We have reported that aPDT mediated by a range of different PS can be strongly potentiated by addition of the non-toxic inorganic salt, potassium iodide^22–25^. We originally hypothesized that the mechanism of action involved one-electron transfer to iodide anion to produce iodine radicals (Type I), but subsequent studies showed that iodide underwent an addition reaction to singlet oxygen (Type II) to produce reactive iodine species and hydrogen peroxide^22^ also producing the stable antimicrobial substance, iodine/tri-iodide. We also demonstrated that iodide potentiation of aPDT could be demonstrated *in vivo*, using cationic functionalized fullerenes + KI in a mouse model of skin abrasions infected with *Acinetobacter baumannii*^25^ or with Rose Bengal + KI in the same mouse model infected with *Pseudomonas aeruginosa*^24^. We also showed that MB combined with KI, excited with red laser was effective treatment in a mouse model of oral candidiasis^26^.

Reasoning that UTIs might be amenable to aPDT given the bladder contains aqueous liquid, we chose the well-known PS and phenothiazinium salt, methylene blue (MB), long FDA-approved, to facilitate early clinical translation. Though there are several papers showing that aPDT using MB is able to kill Gram-negative species such as *E. coli*^27–29^, MB is not highly efficient. Taking into account that bacteria infecting the urothelial lining are likely to be present in biofilms, this would make aPDT even more difficult. We thus filled the bladder with KI, which is highly water-soluble and thus might be more likely to come into close contact with the bacterial surface. This would be important because during light delivery the reactive iodine species produced possess only short diffusion distances.

We previously used stable bioluminescent bacteria and other microbial cells to monitor aPDT *in vivo* in real-time non-invasively^30^. By imaging animals after various doses of light have been delivered, a dose-response curve can be constructed, and imaging over succeeding days allows the chief problem besetting aPDT of infections, regrowth of bacteria after the light delivery has finished, to be monitored. Therefore the goals of this study were to: (1) develop an intact rat model of UTI using bioluminescent UPEC; (2) develop a protocol for aPDT inside the rat bladder; (3) test whether KI could potentiate aPDT with MB.

## Materials and Methods

### Strain, cells and culture conditions

UTI89, a clinical cystitis isolate^31^, was a generous gift from Dr. Patrick Seed’s laboratory. For *in vivo* imaging experiments, UTI89 carrying the complete lux operon (“UTI89-lux”), was selected with 50 μg/ml kanamycin (KM) and 20 μg/ml chloramphenicol (CAM). For all experiments, UTI89-lux was cultured at 37 °C 24 h in Brain Heart Infusion broth (BHI) supplemented with 50 μg/ml KM and 20 μg/ml CAM. Bacteria were then diluted 1/100 into fresh BHI for an additional 24 h at 37 °C before being diluted in PBS.

5637 epithelial cells (derived from a human bladder carcinoma, HTB-9; American Type Culture Collection, Manassas, VA) were cultured in RPMI 1640 medium (Sigma, St. Louis, MO) supplemental with 10% FBS at 37 °C in a water-saturated atmosphere of 5% CO2.

### Antimicrobial PDI *in vitro* studies

Suspensions of bacteria (10(8) cells/mL) were incubated in the dark at room temperature for 30 min with MB (0–10 μM) and added a range of KI concentrations between 0 and 100 mM in PBS. An aliquot of 100 μL was used as the dark control (DC) from each sample; another aliquot (200 μL) was transferred to a 48-well plate and illuminated from the top of the plates at room temperature with 10 J/cm^2^ of red light. The light source we used was the diode laser (RPMC Lasers Inc, MO) from Arroyo Instruments (San Luis Obispo, CA), which emitted red light at a wavelength of 660 nm to deliver 10 J/cm^2^ at an irradiance of 50 mW/cm^2^ as measured with a power meter (Thorlabs, NJ, USA). At completion of illumination (or dark incubation), aliquots (100 μL) were taken from each well to determine CFU. Each aliquot was serially tenfold diluted in PBS to give dilutions of 10^1^–10^6^ times, and 10 μL aliquots of each of the dilutions were spotted onto square BHI agar plates. Plates were potted in triplicate and incubated for 16–18 h at 37 °C in the dark to allow colony formation. Experiments were done at least three times, with reproducible results.

Subconfluent 5637 epithelial cells were seeded in the 96-well plate (5000 cells/well) 24 h prior to 30 min incubation of MB (0–10 μM) and added a range of KI concentrations between 0 and 100 mM in RPMI media. Each group had 4 wells of 5637 epithelial cells. Cell viability was determined by PrestoBlue®assay (Thermo Fisher Scientific, CA, USA) according to the manufacturer’s manual. The experiment was repeated 3 times.

### Animals

Female Sprague-Dawley rats (Charles River Laboratories, Wilmington MA), weighing 275 g to 325 g, were used according to experimental protocol #2015N000073 approved by the Institutional Animal Care and Use Committee at MGH. Experiments were also in accordance with the NIH Guide for the Care and Use of Laboratory Animals, 8th edition

### Bioluminescent imaging

The IVIS® Lumina Series III *in vivo* imaging system (PerkinElmer, Inc., Waltham, MA, USA) was used for bioluminescence imaging before, during, and for up to 16 days after the infection. Using photon-counting mode, an image was obtained by detecting and integrating individual photons emitted by the bacterial cells. Anesthesia was induced with 3% inhaled isoflurane. A grayscale background image of each rat was made, and this was followed by a bioluminescence image of the same region displayed in a false-color scale ranging from red (most intense) to blue (least intense) and superimposed on the grayscale image. The signal from the bioluminescence image was quantified as region of interest (ROI) with absolute calibrated data in photos (s-1 cm-2 sr-1) using the Living Imaging software (Perkin Elmer).

### Rat model of UTI

All animal procedures were performed under anesthesia with 3% inhaled isoflurane. Briefly, anesthetized rats were transurethrally catheterized with a lubricated, sterile 20-gauge angiocatheter without needle. After aspiration of urine in bladder, 2*10^7^ of UTI89-lux suspension was slowly instilled via the angiocatheter into the bladders of rats over 1 min to avoid vesicoureteral reflux. The angiocath prevented voiding for 1 hour and then removed. The results displayed for each experiment were combined from 8 biological replicates.

### Histopathology

Bladders were fixed in 10% neutral buffered formalin for over 48 hours, embedded in paraffin, sectioned (5 μm thick), and stained with H&E and imaged using the Nanozoomer Imager system (Hamamatsu Photonics, Hamamatsu, Japan) with software NDPview (Hamamatsu Photonics, Hamamatsu, Japan).

### Intravesicular aPDT

**Figure 1:**
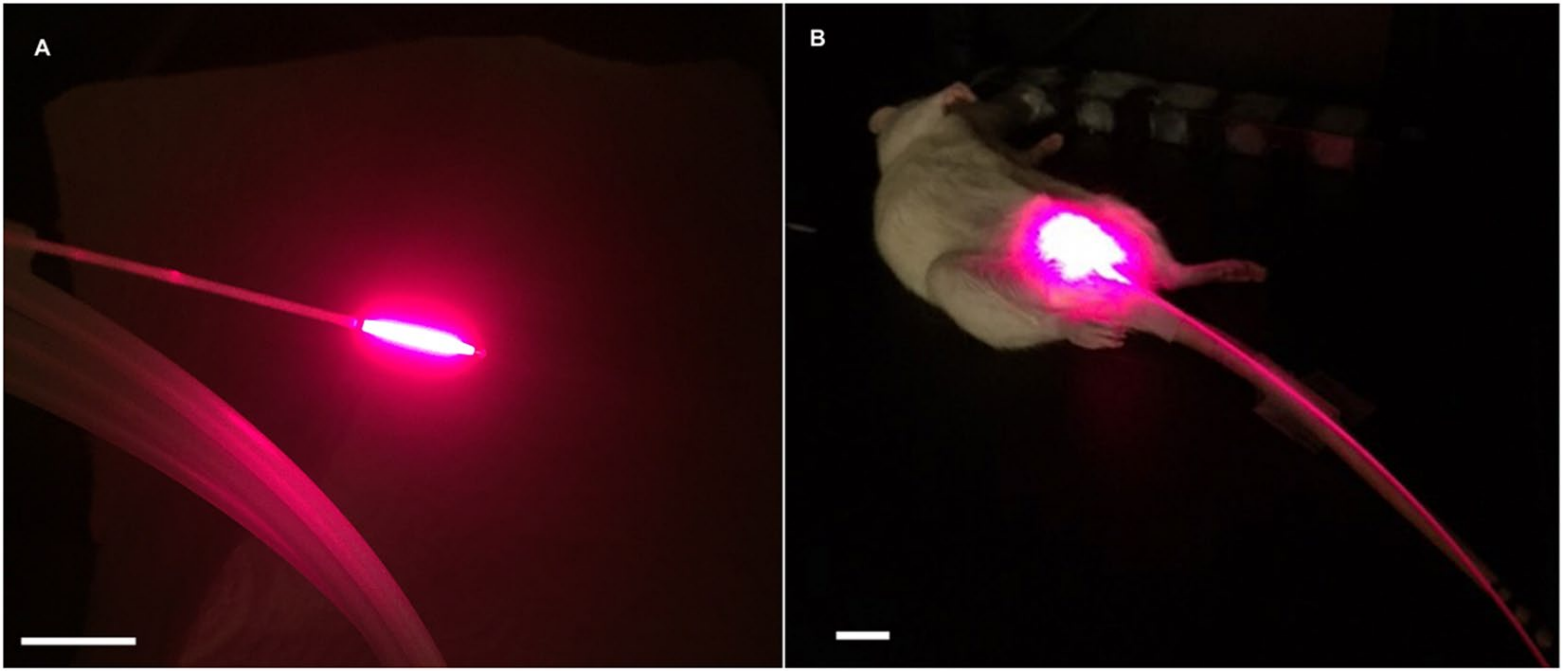
Intravesicular light delivery. Photographs of fiber optic diffusing tip (**A**) and female rat with fiber optic inserted in bladder (**B**).

One hour after infection of the rats, 0.5 mL of MB (100 μM) solution was instilled into the bladder via catheter for 15 mins before light irradiation. At the end of the instillation period, MB solution was withdrawn, then bladders were instilled with a 0.5 mL aliquot of 100 mM KI solution. Control animals were instilled with PBS. For irradiation, the 660 nm laser was coupled into a fused plastic fiber (core diameter 500 µm) with a glass cylindrical diffusing tip (diameter 0.98 mm, length 10 mm) (Medlight, Lausanne, Switzerland). For irradiation of the bladder, the fiber was inserted into the bladder directly and fixed in a central position. Illumination was for 32 minutes. The output power at the end of the fiber was measured using a photometer and calculated with integrating sphere (Thorlabs Inc., NJ, USA). The rat bladder with this volume of liquid was about 1 cm^2^ in surface area^32^. The incident fluence rate at the inner surface of the bladder was determined from the output power divided by the calculated urothelial surface area, assuming the bladder to be spherical^33^. The fluence rate on the bladder surface was 50 mW/cm^2^ and fluences used ranged from 50 J/cm^2^ to 100 J/cm^2^. Preliminary experiments showed that a light fluence of 200 J/cm^2^ delivered at a fluence rate of 200 mW/cm^2^ with MB and KI was toxic to the entire bladder wall. A higher dose of MB (1 mM or 10 mM) or KI (500 mM) could also cause damage to the bladder. For this reason, only a light fluence rate of 50 mW/cm^2^ and a total fluence of 50–100 J/cm^2^ with 100 uM MB and 100 mM KI were used for whole bladder aPDT. Groups of 8 animals were used for each treatment arm. The experimental set-up for whole bladder wall aPDT is shown in Fig. 1.

### Statistics

Differences between means of bioluminescence values in Fig. 3B were compared for significance with one way ANOVA and Tukey’s post-hoc test. Curves in the Kaplan-Meier graph in Fig. 3B were compared by log rank test.

## Results

### *In vitro* studies

**Figure 2:**
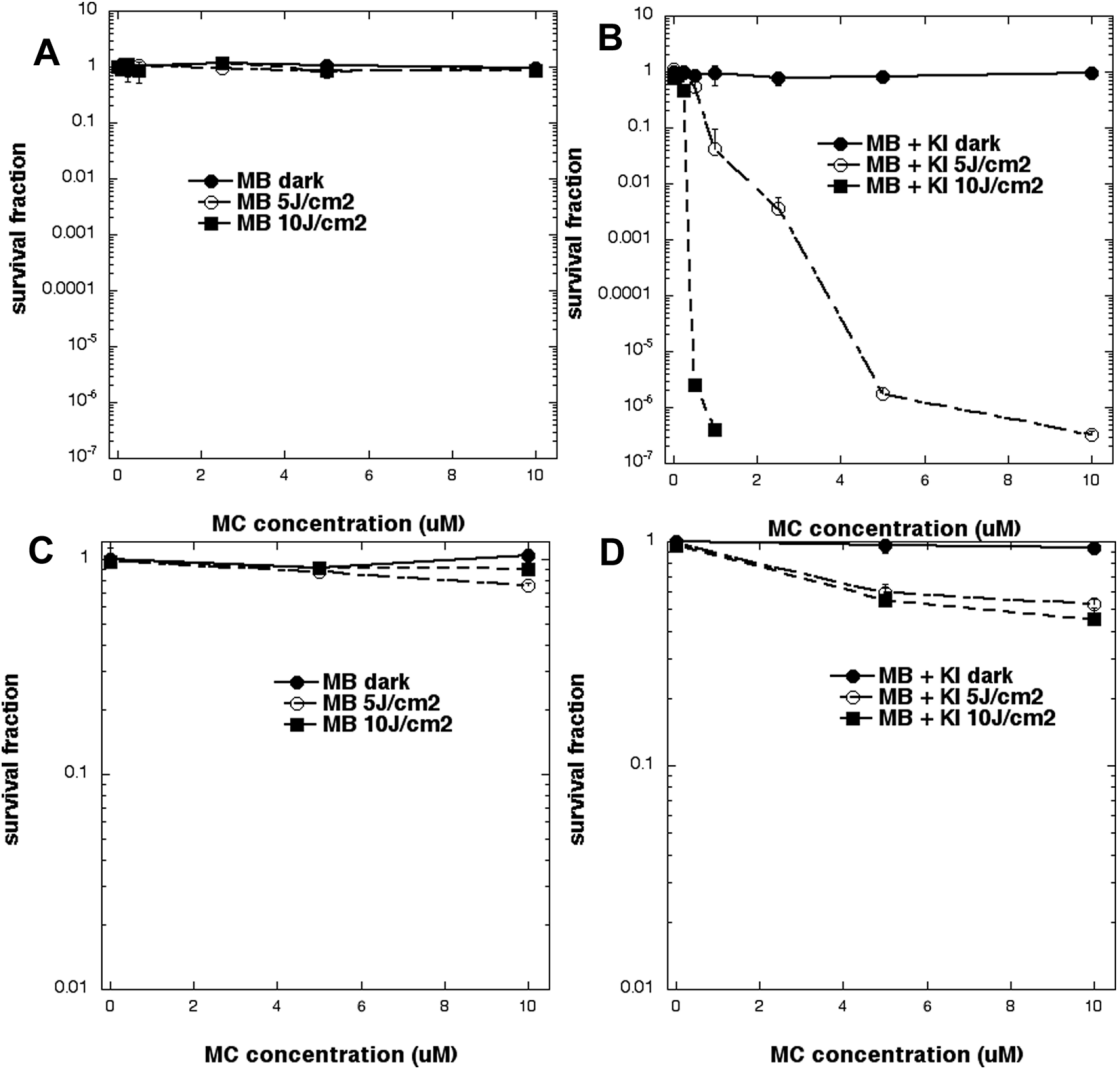
*In vitro* studies with UPEC and urothelial cells. Bacterial cells, UPEC (**A** and **B**) or mammalian urothelial cells (**C** and **D**) were incubated with increasing concentrations of MB without (**A** and **C**) or with (**C** and **D**) 100 mM KI solution and exposed to 5 or 10 J/cm^2^ of  660 nm light.

Figures 2A,B show the *in vitro* aPDT killing of UPEC and the corresponding PDT killing of urothelial cells (Fig. 2C,D) under the same conditions. We compared MB + 660 nm light (A and C) constituting “PDT alone”, and MB combined with 100 mM KI (B and D) as “aPDT + KI”. As can be seen in Fig. 2B UPEC were highly sensitive to PDT + KI with 10 J/cm^2^ producing eradication (>6 log(10) steps) at a MB concentration of only 1 μM, while there was almost no effect under these conditions with traditional aPDT using MB alone + red light (Fig. 2A). With the lower light dose (5 J/cm^2^) eradication was found at 10 μM MB + KI. There was no killing of urothelial cells with MB alone + red light (Fig. 2C) while some cytotoxicity (~40% killing) was observed when KI was added (Fig. 2D), but urothelial killing did not vary greatly when either the concentration of MB or dose of light was increased.

### aPDT for UTI in rat model

We carried out pilot experiments to determine the best inoculation conditions to initiate the rat cystitis but not pyelonephritis. 2*10^7^ of UTI89-lux was chosen as the inoculation cell number according to the literature and preliminary data^31^. With care taken to avoid bladder trauma, the infectious burden was large at day 3, but had gone by day 5 or 6. To demonstrate the value of the MB-PDT + KI combination, we first used a mixture of MB and KI solutions instilled simultaneously. The high MB concentration was not compatible with 100 mM KI solution in PBS; therefore MB was salted out. Subsequently, rats were divided into four groups:(a) absolute control; (b) MB + KI; no light; (c) MB + laser alone; (d) MB + KI + laser (aPDT + KI). Rats were imaged before and after addition of MB/KI, after delivery of 50 J/cm^2^ 660 nm laser and after delivery of 100 J/cm^2^. Rats that lost luminescence immediately after the application of MB and KI were not included. The diffusing optical fiber tip is shown in Fig. 1A; the same fiber inserted in the bladder in Fig. 1B.

**Figure 3:**
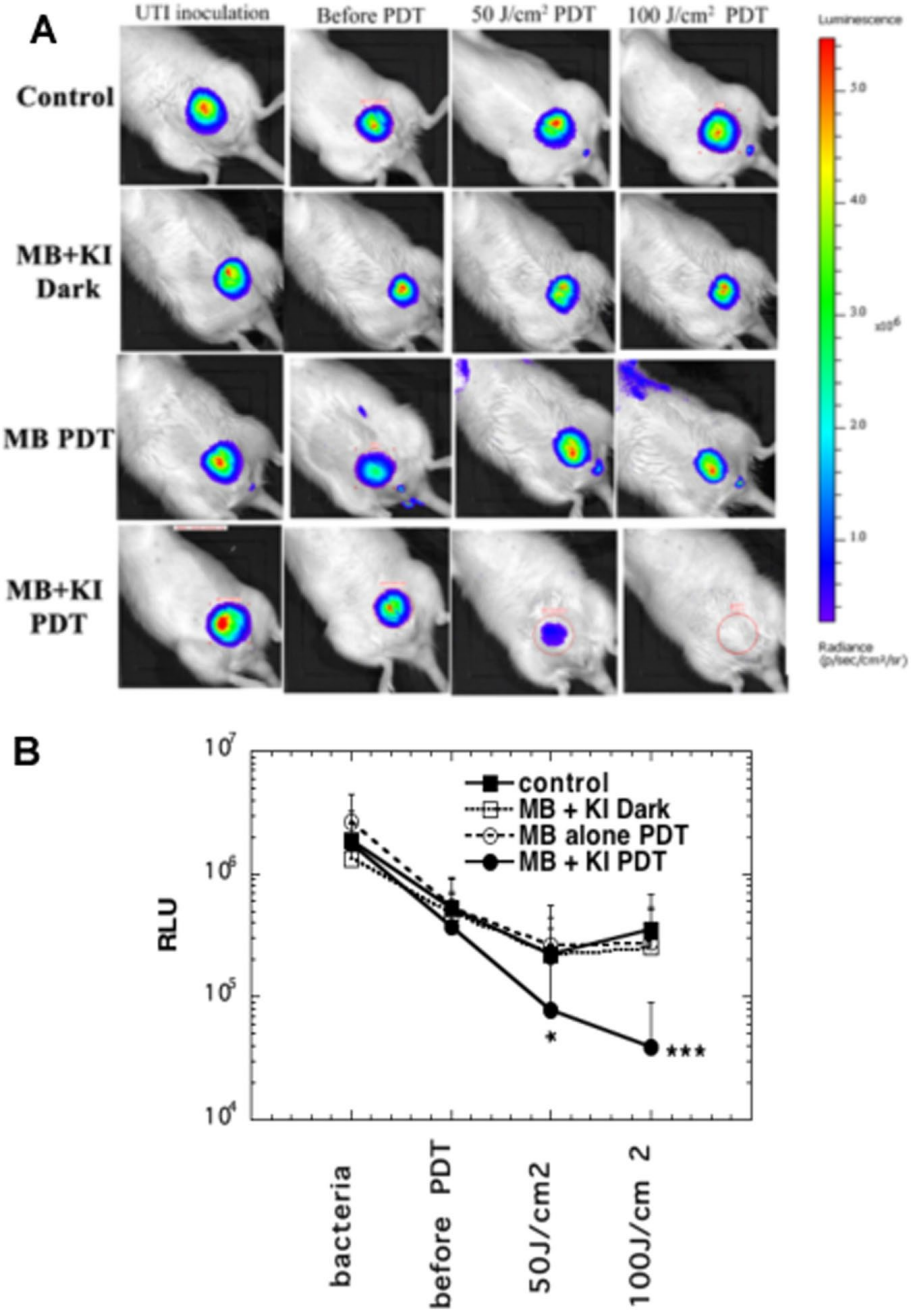
Effectiveness of aPDT on day 0. (**A**) Panel of representative bioluminescent images showing aPDT dose response on the same day of treatment. (**B**) Plot of mean bioluminescence values showing aPDT dose response. Values are means and SD of bioluminescence RLUs from n = 5–9 rats per group. *p < 0.05; ***p < 0.001.

Representative examples of rats from each of these groups are shown in Fig. 3A. Not all the results in the aPDT + KI red laser group were as clear as Fig. 3A (bottom row) but the means + SD data used to plot the graph shown in Fig. 3B take account of the variation in efficiency of individual aPDT sessions. After 100 J/cm^2^ had been delivered, mean luminescence values from the MB + KI PDT group were significantly lower than those from the MB aPDT alone group (p < 0.001).

**Figure 4:**
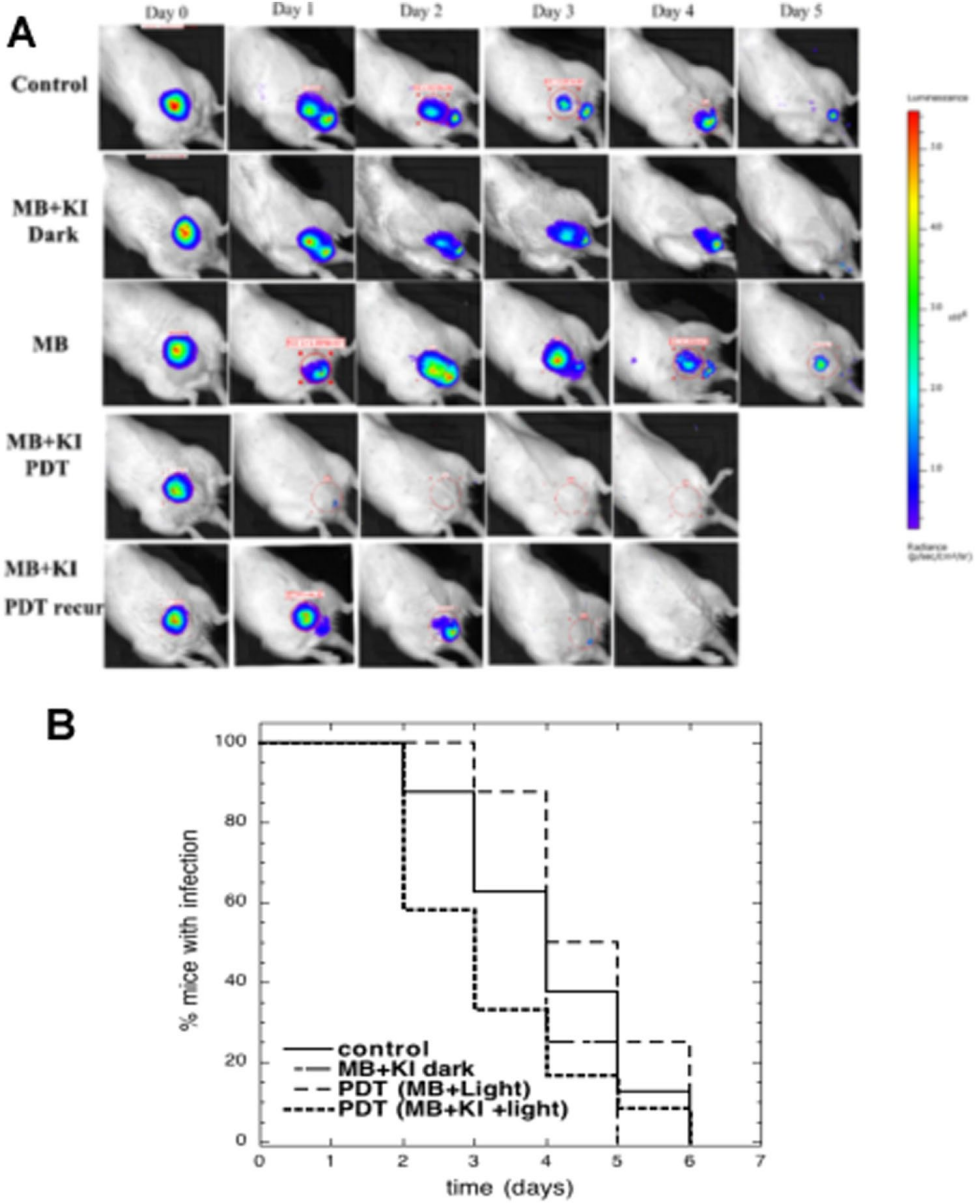
Effectiveness of aPDT over 6 days. (**A**) Representative bioluminescent images over time course of 6 days. (**B**) Kaplan-Meier plot of mice with any remaining bioluminescence signal from the bladder. Control (n = 8); MB + KI dark (n = 8); MB aPDT (n = 8); MB + KI aPDT (n = 12). MB + KI + light curve is significantly different from curves are significantly different from other groups (p < 0.05; log rank test).

Figure 4A shows panels of bioluminescence images captured on day 0 immediately before PDT, and the five succeeding days after aPDT. The rat treated with MB + KI aPDT, shown bottom row Fig. 4A was a complete success (together with 2 other animals). There was no recurrence on day 1 or thereafter. In some rats, bioluminescence did recur the day after treatment though all rats receiving MB + KI aPDT were free of bioluminescence immediately after the 100 J/cm^2^ had been delivered. We therefore made a Kaplan-Meier plot of “% of rats with remaining bioluminescence” as a function of the number of days post-aPDT (Fig. 4B). Using the log rank test, the curves for MB + KI PDT and control were shown to be significantly different (p < 0.05).

### Histology

**Figure 5:**
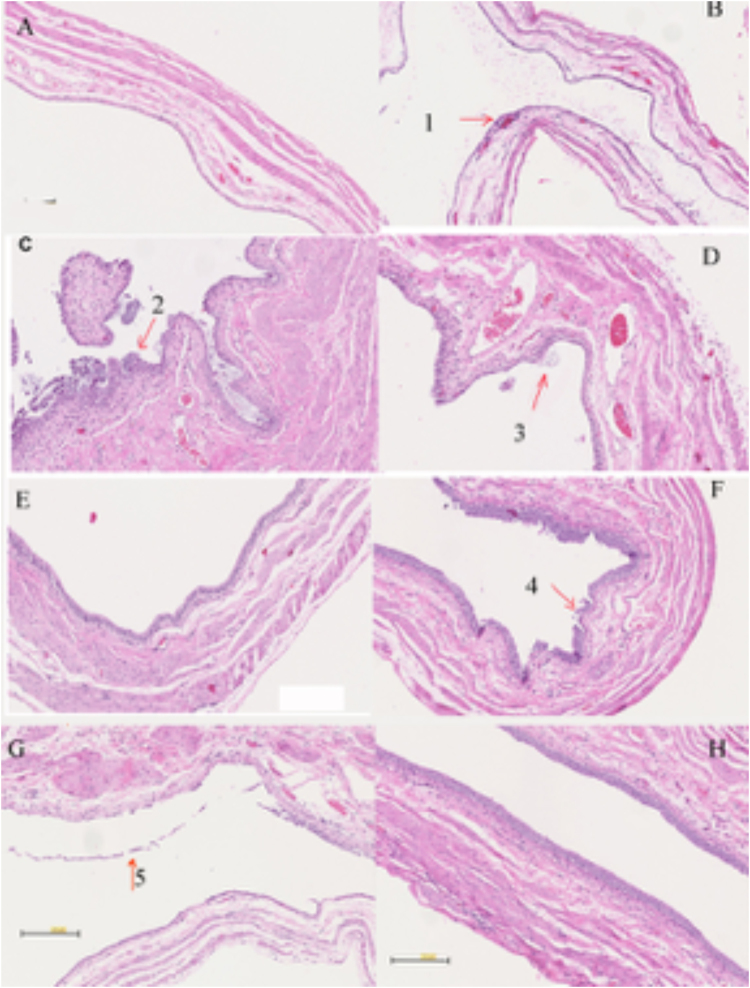
Histology images (H&E stained). (**A**) Normal Bladder Control; (**B**) UTI infection (**C**) MB + KI dark; (**D**) MB PDT; (**E**) MB + KI + PDT; (**F**–**G**) MB + KI + PDT with some injury of surface epithelial cells. G. MB + KI + PDT 2 weeks after inoculation. Bar 200 μm. (Arrow 1, 2 and 3 show intracellular bacterial communities, arrows 4 and 5 show urothelial damage).

Histopathology indicated that acute infected animals mounted a robust acute inflammatory response to infection in their bladders by 24 h post-infection. Representative images of infected bladders from different groups are shown in Fig. 5. The normal (non-infected) bladder is shown in Fig. 5A. Neutrophilic infiltrates are present within the lamina propria and extend into the transitional epithelium in infected bladders (Fig. 5B), MB-PDT treated (Fig. 5C) and MB + KI dark (Fig. 5D) treated animals are also shown. The MB + KI PDT treated animals showed least inflammation and less neutrophil infiltration at 24 h post infection (Fig. 5E). There was some damage of the superficial layer of the urothelium of the bladder wall, but no significant damage to the deeper layers of the bladder wall; Fig. 5G. The results of MB + KI aPDT showed that photodynamic damage was confined to the urothelium of the bladder, without damage to the underlying muscle layer. After 2 weeks of MB-KI aPDT treatment, the urothelium of the bladder wall had a normal appearance. The catheterization process in some animals may cause some damage to the bladder wall during the procedure of inoculation (Fig. 5F).

Histologic analysis also revealed the presence of intracellular bacterial communities (IBC) at the luminal surface of bladders in infected rats, MB/KI dark group and MB-PDI group (Arrows in Fig. B, C and D). IBC are collections of bacteria that serve as intracellular foci for rapid bacterial replication protected from both the host inflammatory response and antibiotics. IBC have previously been shown to develop in the bladders of infected mice, rats and humans^34,35^.

## Discussion

To our knowledge this is the first report of transurethral PDT used to treat infection in vivo in the intact bladder. Use of bioluminescent UPEC allowed establishment of stable infection in the female rat bladder. Bioluminescent monitoring showed that bacteria remained in the bladder for up to five days. Virulence factors specific to uropathogens are needed for bacteria to adhere to urothelium, avoid attack by host cells and form long-lasting biofilms^36^. Type 1 pili act as adhesins and invasins, with specificity for bladder epithelial cells. Fimbriae are also involved in recognition of specific targets on urothelial cells^31,36,37^. UPEC can also form “intracellular bacterial biofilm-like pods” in UTIs^37^, which we observed in this model.

The biofilm habitat adopted by UPEC in UTIs presents a considerable challenge to intravesicular aPDT for bladder infections. Bacteria in biofilms are significantly harder to kill compared to planktonic bacterial cells^38^. Often, killing of only 2 log(10) steps can be achieved with biofilm cells under aPDT conditions where eradication (killing > 6 log(10) steps) can easily be accomplished with planktonic cells. In the present study we used the combination of MB-aPDT with added aqueous KI solution to potentiate bacterial killing. We initially reported that KI with PS can dramatically potentiate bacterial killing during aPDT *in vitro* and *in vivo*^25^, and subsequently discovered that Type 2 ROS (singlet oxygen, ^1^O_2_) produced by photoactivated of Photofrin^22^ or Rose Bengal^24^ interacted with KI to produce antimicrobial iodine species. Potentiation of aPDT by KI was demonstrated in several *in vivo* models of infection, including a mouse model of a 3^rd^ degree burn infected by MRSA^25^, a mouse model of oral infection with *Candida albicans*^26^ and a mouse model of skin infection with *Acinetobacter baumannii*^25^.

There is only one previous report describing aPDT of an experimental bladder infection, by Liu *et al*.^39^. They reported that “charge-conversion polymeric nanoparticles” encapsulating chlorin(e6) could be used as effective antimicrobial -photosensitizers to treat bladder infections, using a murine model involving surgical exposure of the bladder via laparotomy, illumination of the bladder from the outside with 664 nm light, then closure of the surgical incision in the abdomen. While demonstrating successful aPDT of bladder infection, this model does not have the same degree of clinical relevance as our trans-urethral intra-vesicular light delivery method. Most PDT animal models targeting the bladder have been directed towards treating bladder cancer^40,41^.

The problem of regrowth of the bacteria after the cessation of the light delivery is one of the principle drawbacks of using aPDT to treat *in vivo* infections. Since the very short-lived bactericidal ROS are no longer produced after illumination, nothing remains after conventional PDT to suppress bacterial regrowth. The addition of KI to MB-aPDT produces both transient reactive iodine species and also the stable long-lived bactericidal species I_2_/I_3_^−^; this phenomenon may present a partial solution to this dilemma. Repeat treatment after an interval while the inoculum is small would be another approach.

Selectivity in aPDT has traditionally been through use of PS chosen to chemically bind to microbial cells and not bind or be taken up by host mammalian cells. This goal was accomplished by having a large number of cationic groups present in the PS molecule, as microbial cells had more pronounced negative charges on their surfaces than mammalian cells, and the cationic charges helped the PS to penetrate into Gram-negative cells^42^. The use of a short drug-light interval is also proposed to provide selectivity for microbial cells over mammalian cells, as uptake of the PS by the latter is comparatively slow compared to bacteria. In the present case, addition of KI to aPDT + MB provided > 6 logs of killing of UPEC, while killing only 40% of the urothelial monolayer *in vitro*. The outer layer of the urothelium regenerates rapidly^43^, even if the superficial cells are killed by PDT, as evidenced by our *in vivo* experiments (Fig. 5F,G). Sloughing of superficial urothelium containing IBCs may indeed confer a therapeutic advantage^43^ especially with retreatment by aPDT at intervals or even single-dose antimicrobial therapy as a strategy.

In conclusion, the combination of two already FDA-approved, simple chemical compounds (MB and KI) suggests that this novel approach may be suitable for early clinical translation for drug-resistant UTI, which are becoming increasingly problematic in many clinical settings.

aPDT treatment of a UTI in an intact rodent model, has never before been demonstrated, and moreover never monitored in real-time by bioluminescence imaging. Rodent models of bacterial infection are perforce imperfect, as they are notoriously resistant to bacterial infection in general^44^. Nonetheless, these models are a necessary first step in our attempt to develop a clinically practical approach to reducing or eliminating MDR bacterial burden in patients who develop chronic MDR bacterial infection owing to long-term catheterization, neurogenic bladder, and the like. It is our hope to turn the very catheter which can cause bacterial UTI, into a way of delivering aPDT, using currently available FDA-approved reagents, with or without added antibiotic therapy. In this acute cystitis model, we document the first step towards that goal.

### Data Availability

Original data will be made available on request.
